# Single-cell analysis reveals urothelial cell heterogeneity and regenerative cues following cyclophosphamide-induced bladder injury

**DOI:** 10.1038/s41419-021-03740-6

**Published:** 2021-05-05

**Authors:** Xiaomu Cheng, Huadong Lai, Wenqin Luo, Man Zhang, Juju Miao, Weichen Song, Shunpeng Xing, Jia Wang, Wei-Qiang Gao

**Affiliations:** 1 State Key Laboratory of Oncogenes and Related Genes, Renji-Med X Clinical Stem Cell Research Center, Ren Ji Hospital, School of Medicine and School of Biomedical Engineering, Shanghai Jiao Tong University, Shanghai, China; 2 Med-X Research Institute, Shanghai Jiao Tong University, Shanghai, China; 3 Shanghai Mental Health Center, Shanghai Jiao Tong University School of Medicine, Shanghai, China; 4 Department of Critical Care Medicine, Ren Ji Hospital, School of Medicine, Shanghai Jiao Tong University, Shanghai, China

**Keywords:** Cell biology, Cell proliferation

## Abstract

Cyclophosphamide is a commonly used chemotherapeutic drug to treat cancer with side effects that trigger bladder injury and hemorrhagic cystitis. Although previous studies have demonstrated that certain cell subsets and communications are activated to drive the repair and regeneration of bladder, it is not well understood how distinct bladder cell subsets function synergistically in this process. Here, we used droplet-based single-cell RNA sequencing (scRNA-seq) to profile the cell types within the murine bladder mucous layer under normal and injured conditions. Our analysis showed that superficial cells are directly repaired by cycling intermediate cells. We further identified two resident mesenchymal lineages (*Acta2*^+^ myofibroblasts and *Cd34*^+^ fibroblasts). The delineation of cell-cell communications revealed that *Acta2*^+^ myofibroblasts upregulated *Fgf7* expression during acute injury, which activated Fgfr signaling in progenitor cells within the basal/intermediate layers to promote urothelial cell growth and repair. Overall, our study contributes to a more comprehensive understanding of the cellular dynamics during cyclophosphamide-induced bladder injury and may help identify important niche factors contributing to the regeneration of injured bladders.

## Introduction

The urothelium is a multilayered epithelium that serves as a crucial barrier between the blood and urine. Mature urothelium is composed of different cell types that are arranged into basal, intermediate, and superficial cells, on the basis of cell location and morphology^1,2^. Under normal conditions, the urothelium is very quiescent and undergoes cell turnover every ~200 days in rodents^3,4^. However, in response to acute injuries caused by exposure to chemicals (e.g., cyclophosphamide (CPP)^5–7^ and protamine sulfate^8,9^), uropathogenic bacteria^7–9^, or surgical damage^10^, the urothelium can initiate a rapid repair and regeneration process. We and other groups have characterized the cellular origin of urothelial regeneration using cell lineage tracing studies^5–7,9–11^. Basal progenitors are capable of self-renewal and serve as precursors during long-term injury and regeneration, while intermediate cells undergo more incomplete cytokinesis to increase ploidy and serve as the direct progenitors for repairing superficial cells. Furthermore, different groups have attempted to elucidate the tissue niche that regulates urothelial regeneration. For example, Shin et al. reported that Hedgehog/Wnt feedback between urothelial and stromal cells supports regenerative proliferation of basal epithelial stem cells in the bladder^8^.

Despite these efforts, a comprehensive understanding of cellular heterogeneity and crosstalk during bladder regeneration is still lacking. Recent advances in single-cell RNA sequencing (scRNA-seq) technologies have enabled us to study the transcriptional dynamics at a single-cell resolution and to explore various processes, such as cell cycle regulation^12–14^ and cell-cell communications^15–18^. Here, we used droplet-based scRNA-seq to decipher the cellular dynamics of bladder mucosa and the signals driving the proliferation of urothelial progenitor cells after CPP-induced injury.

## Results

### Single-cell transcriptomic profiling of injured and uninjured mice bladder mucosa

**Fig. 1:**
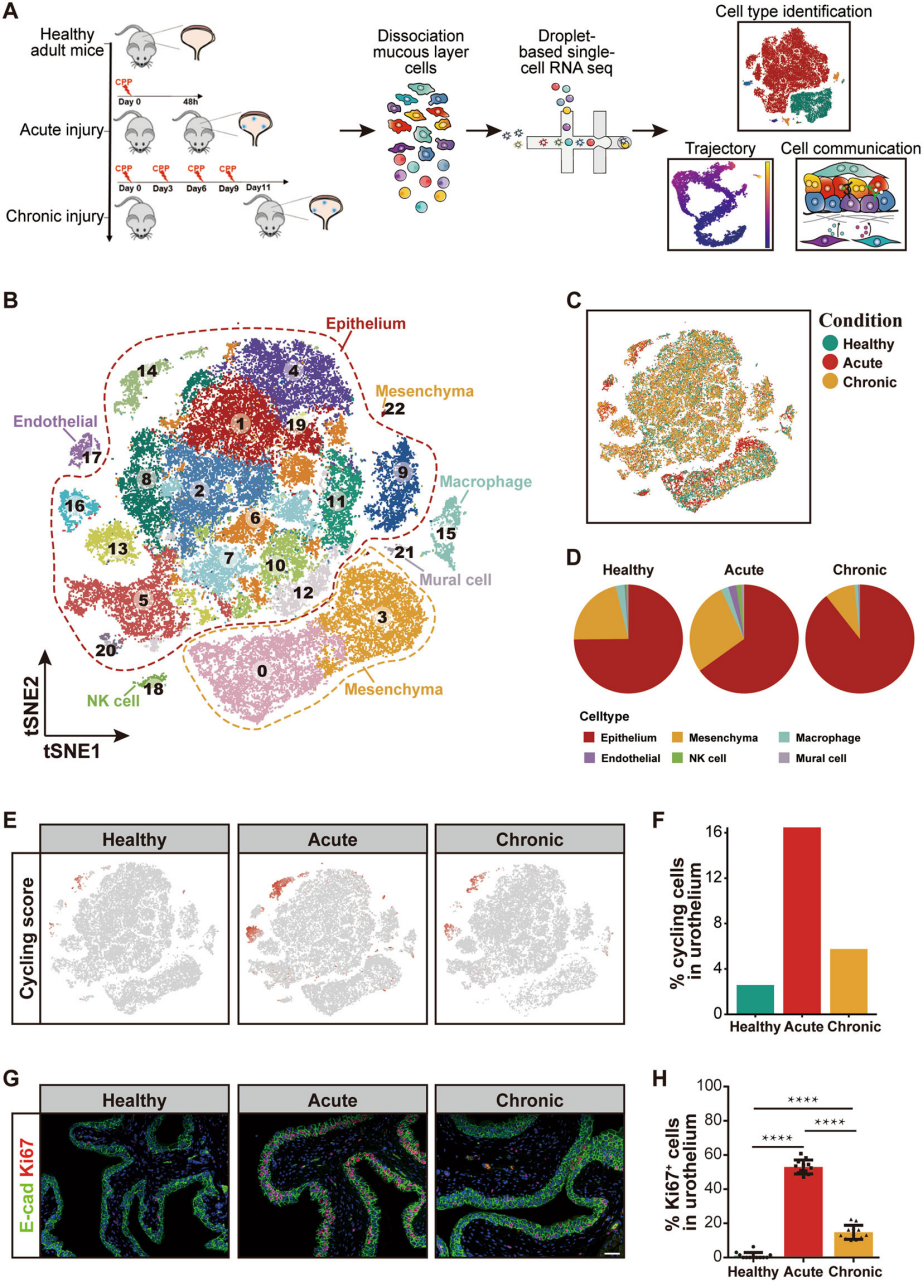
Single-cell profiling revealed cell heterogeneity in mice bladder mucosa layer under healthy and injury conditions. **A** Overview of the experimental workflow. **B** t-Distributed stochastic neighbor embedding (t-SNE) visualization of all cells from healthy, acute injury and chronic injury conditions. Cells are colored by clusters. **C** t-SNE visualization annotated by injury conditions. **D** The proportion of each cell type under each condition. **E** t-SNE visualization of cells from each condition colored by cycling score. **F** Percentages of cycling cells in urothelium for each condition. **G** Fluorescent immunostainings from the indicated conditions show nuclei (DAPI) in blue, E-cad in green, and Ki67 in red (scale bar 50 microns). **H** Bar plot shows the percentage of Ki67 positive cells in urothelial cells (*n* = 12 per condition, mean with SD, *t*-test, ****p < 0.0001).

To systematically study cell heterogeneity and dynamics of bladder mucosa under healthy and injured conditions, we modeled acute and chronic bladder injury using CPP as previous studies reported^19–21^. Then, we performed scRNA-seq for bladder mucosa collected from healthy adult mice as well as acutely and chronically injured adult mice (*n* = 4–6 mice per condition) (Fig. 1A). Of note, two biological replicates were used for each condition (Fig. S1A, B).

After filtering out low-quality cells, we captured a total of 50,404 cells for further analysis, with an average of 2011 unique genes detected per cell. We integrated cells from the three conditions using the *IntegrateData* function in Seurat to remove any batch effect. We identified 23 cell clusters and grouped the cell clusters into epithelial cells (expressing *Cdh1*, *Epcam*, and *Gata3*), mesenchymal cells (expressing *Vim*, *Sparc*, and *Dcn*), macrophage cells (expressing *Cd68* and *Cd83*), endothelial cells (expressing *Pecam1*), mural cells (expressing *Cspg4*)^22^, and natural killer (NK) cells (expressing *Hcst* and *Ltb*)^23^ (Fig. 1B and Fig. S1C). Of these cells, epithelial and mesenchymal cells comprised most of the cells in the bladder mucosal layer (Fig. 1C, D).

We next asked whether single-cell data could reflect the features of the regenerating urothelium adequately. For this purpose, we assessed the cycling cell population in single-cell datasets, given the fact that the CPP-induced injury/regeneration process of bladder is accompanied by abundant proliferative cells^5–7^. We observed that proliferating cells were mostly belonged to the epithelial cell types, and that the proportion of proliferating urothelial cells was highest in the acutely injured sample (Fig. 1E, F). Consistently, similar results were seen with immunofluorescence assays against proliferative cell marker Ki67 on sections from healthy, acutely injured, and chronically injured adult mice bladder (Fig. 1G), which showed that the mucosal layer of mouse bladder after acute injury contained the most Ki67^+^ proliferative cells (Fig. 1H). Taken together, our single-cell datasets reflect the proliferation dynamics of healthy and injured bladder samples and can be used for the following analysis.

### Characterization of urothelial cell heterogeneity

**Fig. 2:**
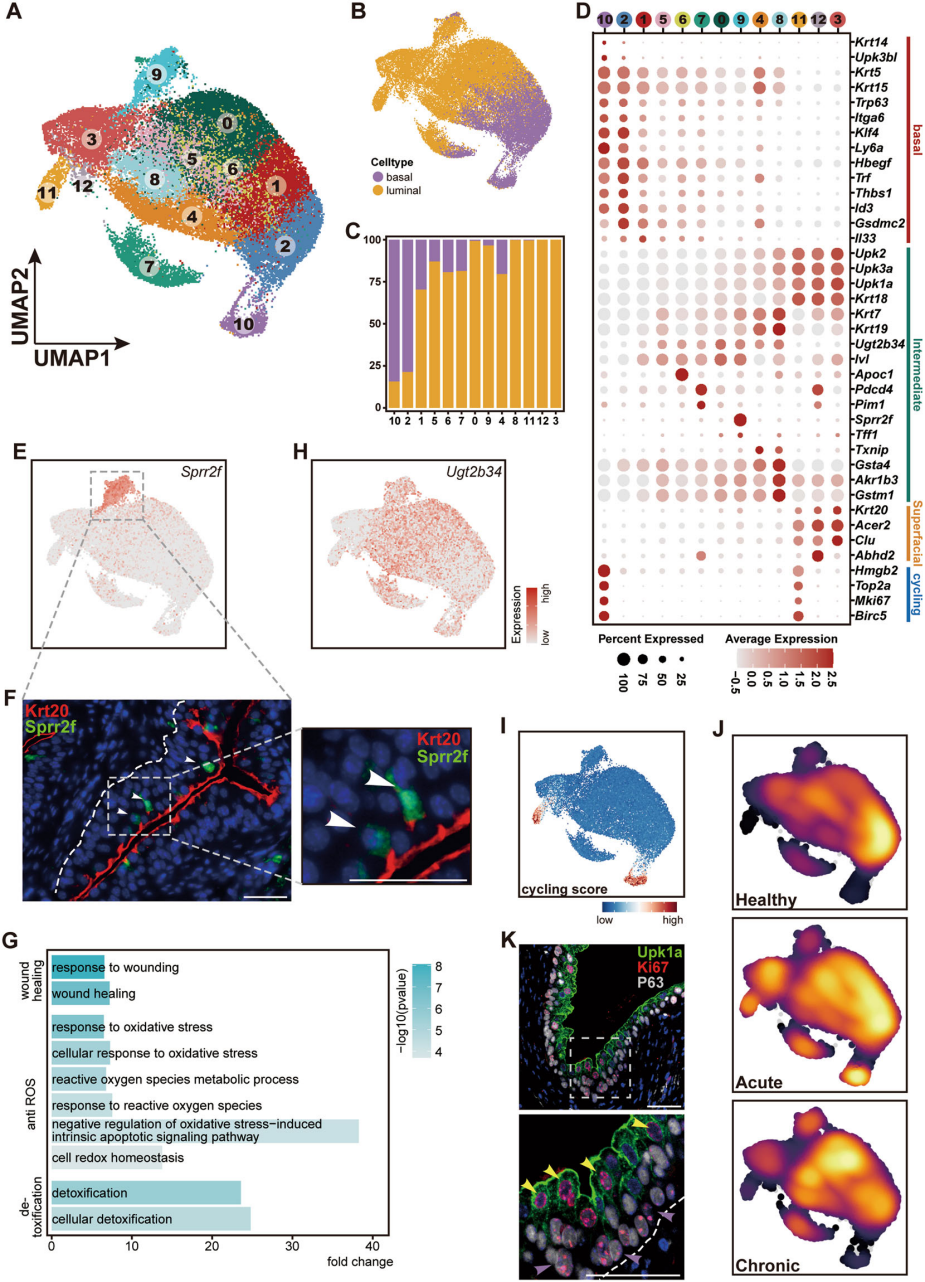
Cell diversity within the urothelium lineage. **A** UMAP visualization of sub-clustered urothelial cells. **B** UMAP visualization of urothelial cells colored by basal subset and luminal subset. **C** Bar plot show the proportion of basal subset and luminal subset in each cluster. Cell subsets are labeled with same color code as in **B**. **D** Dot plot of marker genes in each cluster. **E** Expression of *Sprr2f* projected onto the UMAP. Cells are colored by gene expression. **F** Immunostainings of Krt20 (in red) and Sprr2f (in green) on section of healthy bladder, showing the location of *Sprr2f*^+^ cells in intermediate layer (scale bar 50 microns). White arrowheads point to Sprr2f-positive intermediate cells. **G** GO analysis of genes differentially expressed by cells in *Sprr2f*^+^ subtype intermediate cells. **H** Expression of *Ugt2b34*, a novel signature gene for intermediate subset, projected onto the UMAP. **I** UMAP visualization of urothelial cells colored by cycling score. **J** UMAP visualization of urothelial cells colored by embedding density of cells from three conditions. **K** Immunostainings of Ki67 (in red), Upk1a (in green) and P63 (in gray) on section of acute injured bladder (scale bar 50 microns). Yellow arrowheads point to cycling intermediate cells and purple arrowheads point to cycling basal cells.

Next, we sought to analyze epithelial heterogeneity in healthy and injured conditions by performing a second round of cluster analysis on urothelial cells. This analysis identified a total of 13 sub-clusters of urothelial cells (Fig. 2A). To group and annotate these urothelial cell subsets, we calculated expression scores by summarizing the mRNA fraction per cell of genes associated with the features of basal and luminal cells (i.e., intermediate and superficial cells) as well as the cell cycle state. To define basal/luminal identity, we first determined gene sets associated with basal/luminal phenotypes, including cytokeratin genes, uroplakin genes, and *Trp63*, by calculating their *Pearson* correlation (Fig. S2A). We then scored each cell for their expression levels of basal/luminal gene sets (Fig. 2B and Fig. S2B). This analysis revealed two clusters (cluster 2 and 10) with prominent basal phenotypes (Fig. 2C and Fig. S2B). Accordingly, cells of these two clusters highly expressed basal epithelial genes such as *cytokeratin-14* (*Krt14*) and *cytokeratin-5* (*Krt5*) (Fig. 2D). In addition, we found that they also highly expressed genes associated with stemness (*Ly6a*, *Klf4*, and *Id3*) and ion transport (*Trf*) as well as genes mediating cell-to-matrix interactions, such as *Thbs1* (Fig. 2D). Of the luminal clusters, clusters 3 and 12 highly expressed *Krt20* and were thus defined as superficial cells (Fig. 2D). In addition to *Krt20*, superficial cells also preferentially expressed genes that are associated with lipid metabolism, such as *Acer2* (Fig. 2D). The remaining luminal cells were thus referred to collectively as intermediate cells. Of interest, intermediate cells exhibited moderate expression of basal markers such as *Krt5* and *Krt15* (Fig. 2D).

We additionally obtained cluster-specific signatures. For example, the two superficial subsets were heterogenous, as cluster 12 selectively expressed *Abhd2*, an acylglycerol lipase that catalyzes the hydrolysis of endocannabinoid arachidonoylglycerol from the cell membrane^24,25^ (Fig. 2D). Cluster 6 of intermediate cells selectively expressed *Apoc1*, which encodes protein that plays a central role in the metabolism of high-density and very low-density lipoproteins^26^. Cluster 9 specifically expressed the genes *Sprr2f* and *Tff1* (Fig. 2D and E), both of which are reported to be linked to epithelial repair^27–29^. Gene ontology (GO) analysis of cluster 9 signatures supported that *Sprr2f*^+^ intermediate cells in bladder might play a function in protecting urothelium from injury, as evidenced by significantly enriched terms associated with wound healing, anti-reactive oxygen species (ROS), and detoxification (Fig. 2G). The *Sprr2f*^+^ intermediate cells were confirmed in the intermediate layer by immunofluorescence staining of healthy bladders (Fig. 2F).

Subsequently, we sought to determine whether intermediate cells have their own signatures, as intermediate cells are usually classified by a combination of cell lineage markers, such as Krt5^-^P63^+^Krt20^-^. We found that *Ugt2b34*, a gene associated with glycosylation, was preferentially expressed in intermediate cells but not basal and superficial cells, showing for the first time that intermediate cells have their own identity (Fig. 2H).

We then analyzed cycling cell populations, which were found in both basal (cluster 10) and luminal compartments (cluster 11) (Fig. 2I). The cycling basal cells (cluster 10) highly expressed *Krt14* (Fig. 2D), in line with a recent study^6^. Of note, the cycling luminal cells (cluster 11) exhibited traits resembling superficial cells (e.g., *Acer2* and *Clu* expression), indicating their close relationship. Both the cycling populations were highly enriched in the acutely injured condition (Fig. 2J and Fig. S2C). Further immunofluorescence analysis confirmed this observation, showing that Ki67^+^ cells could be detected in both the basal and luminal layers after acute injury (Fig. 2K). Collectively, scRNA-seq enabled us to capture transcriptionally distinct urothelial cell subpopulations and to highlight the cycling cell subsets within both basal and luminal compartments.

### Basal and intermediate cells display distinct cell cycle progression patterns

**Fig. 3:**
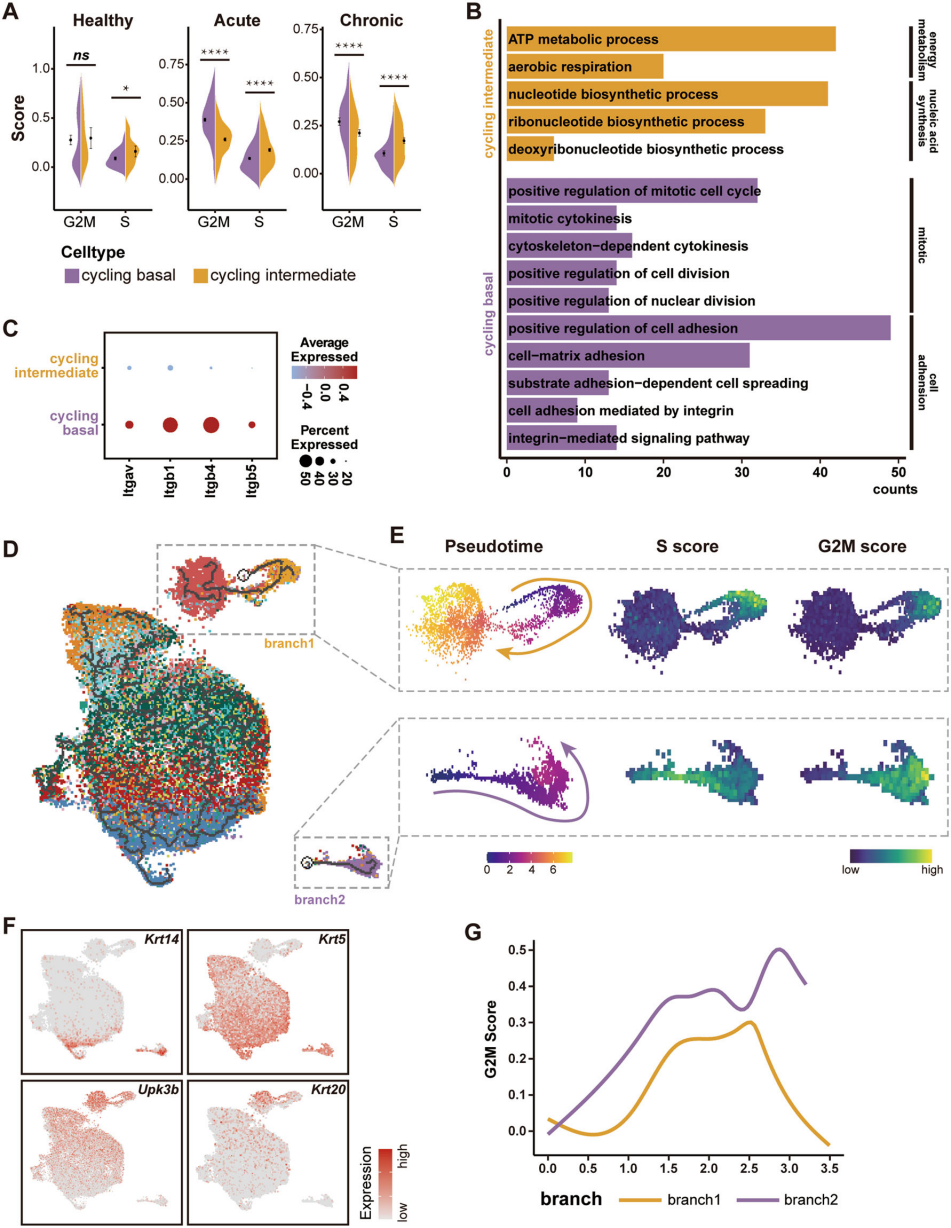
Cell cycle discrepancy between basal and intermediate cells. **A** Violin plots show the G2M-score and S-score of cycling basal cells and cycling intermediate cells in each condition. *p = 0.0161, ****p < 0.0001. Unpaired two-sided t test. **B** GO analysis of genes differentially expressed by cells in cycling basal and cycling intermediate subset. **C** Dot plot show expression of *Itgav*, *Itgb1*, *Itgb4* and *Itgb5* in two cycling subsets. The color represents scaled average expression of marker genes in each subset, and the size indicates the proportion of cells expressing marker genes. **D** Trajectory of urothelial cells reconstructed by Monocle 3. Clusters are labeled with same color code as in Fig. 2A. **E** Pseudotime, S-score and G2M-score of branch1 (cycling intermediate to superficial) and branch2 (cycling basal). **F** Expression of signature genes for basal, intermediate and superficial subset projected onto the UMAP. **G** Kinetics plot showing G2M-score of cycling cells in branch1 and branch2 across pseudo time.

We previously showed that urothelial regeneration is mediated by distinct division modes of cycling basal and intermediate cells^7^. Basal cells undergo more complete cell divisions for self-renewal, while cycling intermediate cells are unable to complete cytokinesis leading to the generation of ploidy^7^. Notably, the heterogeneous cell cycle phases, corresponding to the two well-known checkpoints, can be distinguished by sc-RNA data^12^. We thus assessed the signatures of different cycling phases (i.e., the G1/S and G2/M phases) in cycling basal and luminal cells over the course of the disease. We consistently observed that the G2/M score of proliferating basal cells was significantly higher than that of proliferating intermediate cells in both chronic and acute injury (Fig. 3A and Fig. S3A). Further GO analysis also supported that cycling basal cells underwent a more complete division, as shown by enrichment of the GO terms associated with cell or nuclear divisions, which were absent in cycling intermediate cells. Interestingly, this analysis highlighted that cycling basal cells were significantly enriched for GO associated with cell adhesion (Fig. 3B). Consistently, we found that several cell adhesion components such as *Itgb1*, *Itgav*, and *Itgb5* were strongly expressed in basal cells but not intermediate cells (Fig. 3C and Fig. S3B). This finding thus provided insight into the cell cycle discrepancy between basal and intermediate cells, as downregulation of cell adhesions has been widely reported to affect normal cell division^30^. Taken together, our single-cell analysis confirmed the cell cycle discrepancy between basal and intermediate cells and suggested that the discrepancy might be due to the difference in cell adhesion between the two subsets of cycling cells.

### Cycling intermediate cells are the progenitors of superficial cells

We previously proposed that cycling intermediate cells could serve as the direct progenitors of superficial cells^7^. To verify this hypothesis, we next investigated the lineage relationship between superficial and other urothelial cell subsets. We used the Monocle 3 algorithm^31–33^ to reconstruct the pseudotime of urothelial cell differentiation. This analysis revealed that cycling basal cells and cycling intermediate cells represented two branches of urothelial states (Fig. 3D, E). Importantly, cycling intermediate cells were tightly grouped with superficial cells, demonstrating their close relationship (Fig. 3D, F). In contrast, most cycling basal cells were projected as separate clusters or states, implying that they may undergo divisions for self-renewal rather than differentiation (Fig. 3D, F). The trajectory analysis also showed that cycling basal cells undergo a cell cycle transition from the early cell cycle phase (G1/S phase) toward the late cell cycle phase (G2/M phase), while cycling intermediate cells seem to directly differentiate into superficial cells without undergoing the G2/M phase (Fig. 3E, G). These data together support our previous view that cycling intermediate cells can directly differentiate into superficial cells through an uncomplete cell cycle.

### Mesenchymal cells serve as potential upstream signals of urothelial regeneration

**Fig. 4:**
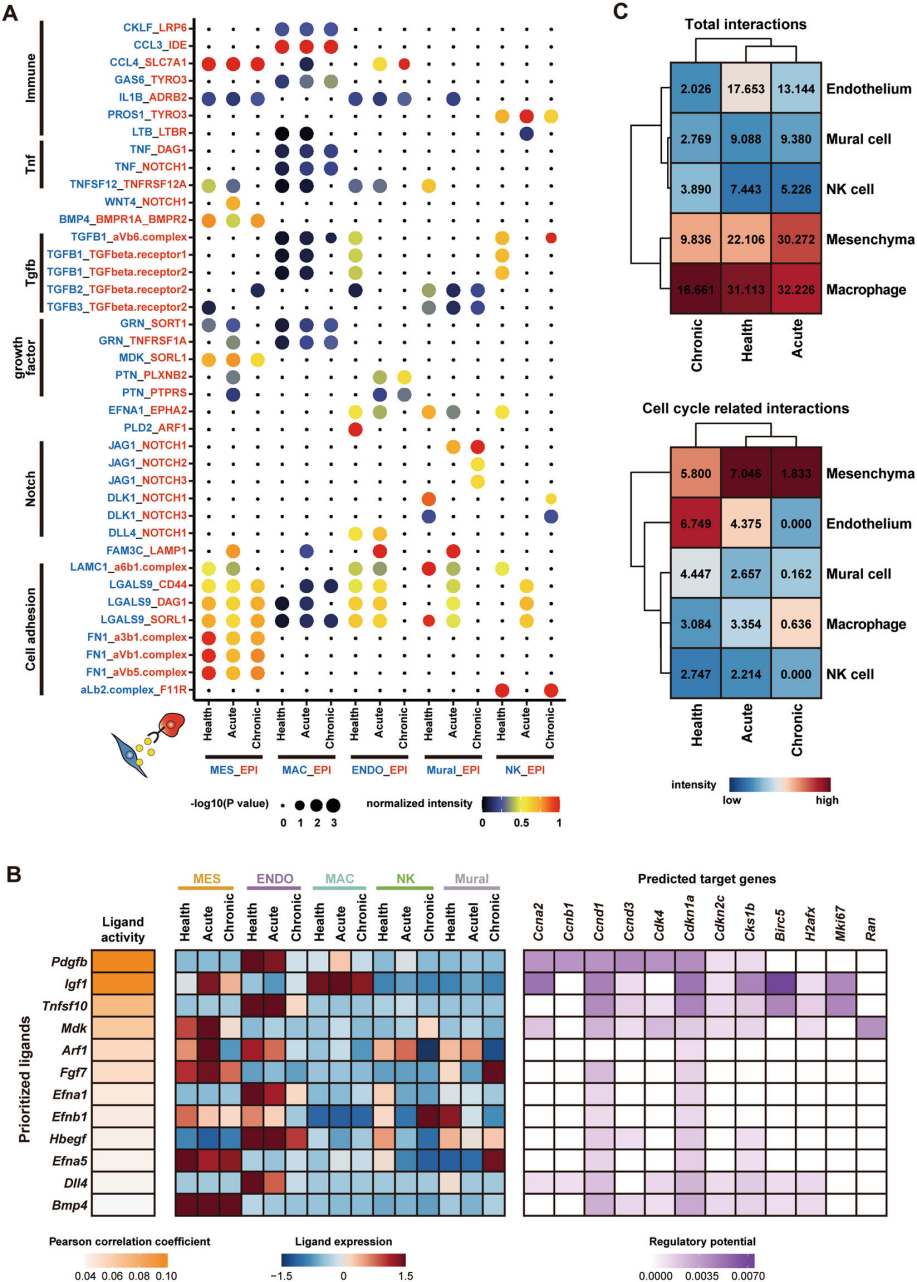
The signaling Niche of urothelium regeneration. **A** Dot plot show top5 interactions from each non-epithelial cell types to epithelial cells predicted by CellphoneDB. **B** Schematic representation of the NicheNet analysis of ligands’ influence on cell cycle entry. Ligands were predicted by CellphoneDB and the top12 potential ligands were showed. Left, heatmap show the ligands activity. Middle, heatmap show the average expression of ligands in each cell type under each condition. Right, heatmap show the potential target genes and the regulatory potential of each activated ligands. **C** Heatmap show the sum of intensity of all communications from non-epithelial cells to urothelium (top), and communications predicted to influence cell cycle entry by NicheNet algorithm (bottom). The numbers showed on the heatmap were the sum of intensity.

Our next aim was to identify the niche mediating urothelial regeneration. For this purpose, we applied CellPhoneDB to construct a putative cell-cell communication network between urothelial and non-epithelial cells, including mesenchymal, macrophage, endothelial, NK, and mural cells^15,34^. This analysis revealed that the homeostasis and regeneration of urothelial cells could be influenced by different microenvironmental cell types. For example, we observed pronounced Bmp4 and Mdk signaling between urothelial and mesenchymal cells in both healthy and injured conditions (Fig. 4A). Macrophages were predicted to secrete cytokines such as *Ccl3*, *Ccl4*, and *Tnf*, which showed robust interactions with urothelial cells (Fig. 4A). Mural cells were predicated to communicate with urothelial cells through Jag1/Notch signaling, especially after chronic injury (Fig. 4A).

We next sought to assess which ligands could exert transcriptional influence on cell cycle entry of urothelial cells. The NicheNet algorithm^35^ was used to predict ligand-target regulatory potential during urothelial regeneration using a cell-cycle gene set as potential pathway targets (Table S3). Our analysis demonstrated that *Mdk*, *Fgf7*, *Bmp4* and etc. could play a significant role in the regulation of genes associated with the cell cycle activity of urothelial cells (Fig. 4B). Interestingly, we found that most of the ligands predicted to promote proliferation of urothelial cells were highly expressed in mesenchymal cells and endothelial cells, suggesting that these two stromal cell types are most likely to promote the regeneration of urothelial cells (Fig. 4B). To further study the potential of each cell type on urothelial regeneration, we then calculated the sum of intensities of total communications from non-epithelial cells to urothelium, as well as the intensities of the communications predicted to influence cell cycle entry with the NicheNet algorithm. We found that, after injury, the interaction intensity from mesenchymal cells to urothelium is much stronger than that from endothelial, mural, and NK cells. In addition, the intensity of interactions that influence cell cycle entry from mesenchymal cells to urothelium is much stronger than that from other cell types after injury (Fig. 4C). These findings thus suggest that mesenchymal cells might exert the most influence on urothelial regeneration, in line with previous studies that examined the role of mesenchymal cells in bladder regeneration^5,8–10^

### Characterization of mesenchymal cell heterogeneity

**Fig. 5:**
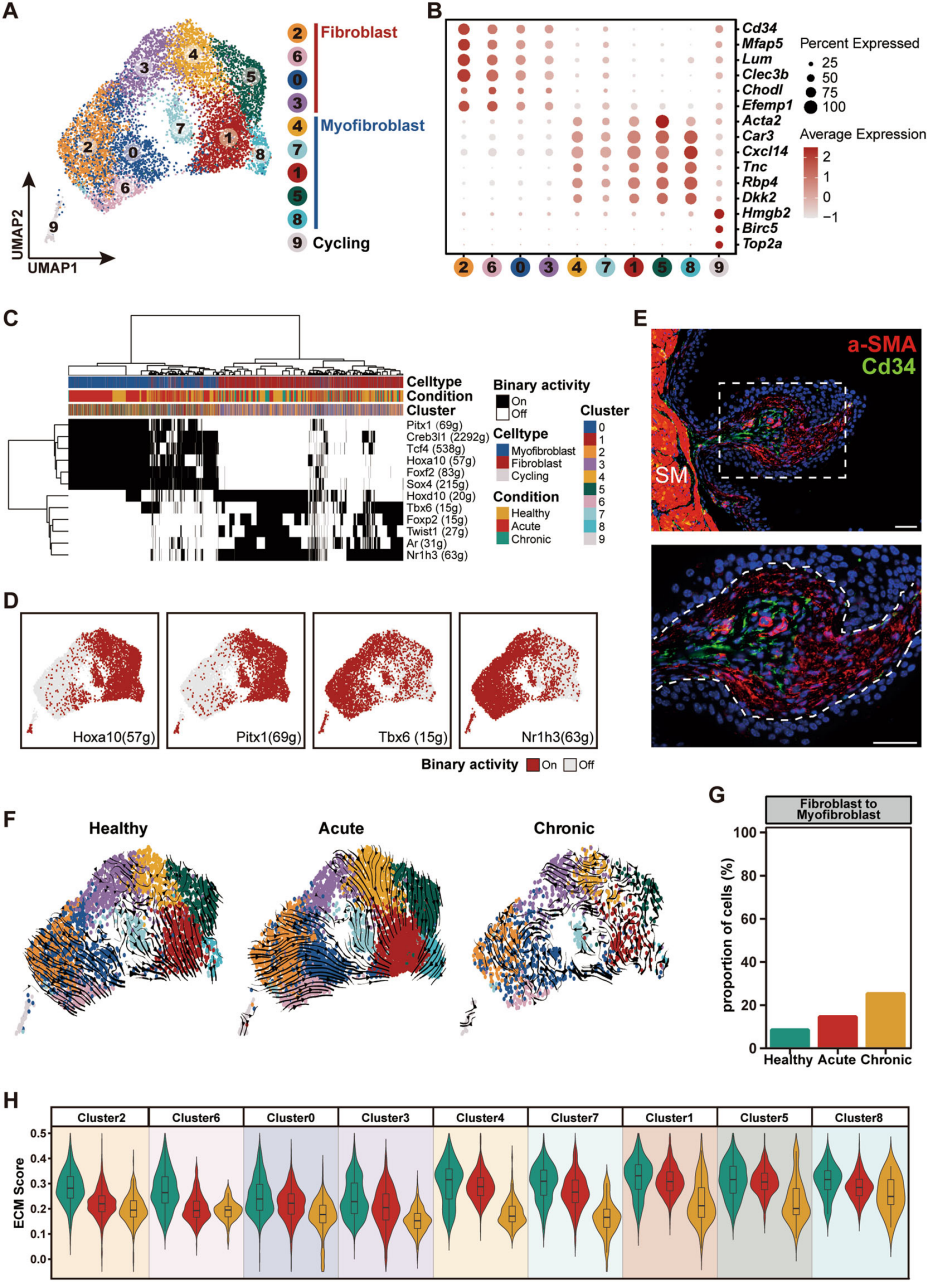
Cell diversity within the mesenchymal lineage. **A** UMAP visualization of subclustered mesenchymal cells in three conditions. **B** Dot plot shows expression of signature genes for fibroblast, myofibroblast and cycling subset in each cluster. The color represents scaled average expression of marker genes in each cluster, and the size indicates the proportion of cells expressing marker genes. **C** Heatmap of SCENIC binary regulon activities shows the distinct regulon activity between myofibroblast and fibroblast. **D** Binary activity of representative regulons for fibroblast and myofibroblast subset projected on UMAP. **E** Immunostainings of a-SMA (in red) and Cd34 (in green) on section of healthy bladder show the existence of myofibroblast and fibroblast, and their location (scale bar 50 microns). **F** RNA velocity of mesenchymal cells under each condition. Clusters are labeled with same color code as in **A**. **G** Bar plot show the proportion of fibroblast cells which can transform to myofibroblasts. **H** Violin plots show the ECM-score in each cluster under each condition. Condition are labeled with same color code as in **G**.

The above analysis highlighted the potential role of mesenchymal cells in bladder repair. To gain a more detailed understanding of bladder mesenchymal cells, we next explored their heterogeneity by performing a second round of clustering analysis. Using marker genes determined from other literature^22,23,36,37^, we characterized two major subtypes corresponding to fibroblasts (*Cd34*^+^*Mfap5*^+^; clusters 0, 2, 3 and 6) and myofibroblasts (*Acta2*^+^*Car3*^+^; clusters 1, 4, 5, 7, 8) (Fig. 5A, B). This analysis identified a population of cycling mesenchymal cells on the basis of the expression of proliferation markers *Hmgb2*, *Birc5*, and *Top2a* (cluster 9) (Fig. 5A, B). To further dissect the transcriptional difference between fibroblasts and myofibroblasts, we analyzed their transcription factor (TF) regulons using SCENIC^38^. This analysis identified a potential TF regulon underlying transcriptional divergence between fibroblasts and myofibroblasts. Fibroblasts preferentially upregulated *Twist1*, *Tbx6*, and *Nr1h3*, while myofibroblasts upregulated *Hoxa10*, *Pitx1*, and *Tcf4* regulons (Fig. 5C, D). We then performed immunofluorescence analysis and found that *Acta2*^+^ myofibroblasts were located in the upper lamina layer near the urothelium, while *Cd34*^+^ fibroblasts were located in the deeper lamina layer (Fig. 5E).

Chronic injury has frequently been associated with tissue fibrosis^39^, which is defined by the accumulation of excess components of the extracellular matrix (ECM). In addition, myofibroblasts play a critical role in tissue fibrosis, and a transition from fibroblasts towards myofibroblasts has been reported to be the underlying cellular mechanism of tissue fibrosis^39–44^. We next asked whether our chronic injury model can induce bladder fibrosis. Modeling cellular state transition using RNA velocity did not capture an obvious transition tendency from fibroblasts towards myofibroblasts (Fig. 5F, G). We also assessed whether serial CPP treatment can induce overproduction of ECM by measuring ECM expression scores that included collagens, glycoproteins, and proteoglycans, in accordance with a recent study^45,46^. This analysis showed that serial CPP treatment significantly downregulated the gene expression levels of ECM components in both fibroblast and myofibroblast subsets (Fig. 5H). All together, these observations suggest that serial CPP treatment in our study did not appear to induce bladder fibrosis.

### Myofibroblasts play an important role in urothelial regeneration

**Fig. 6:**
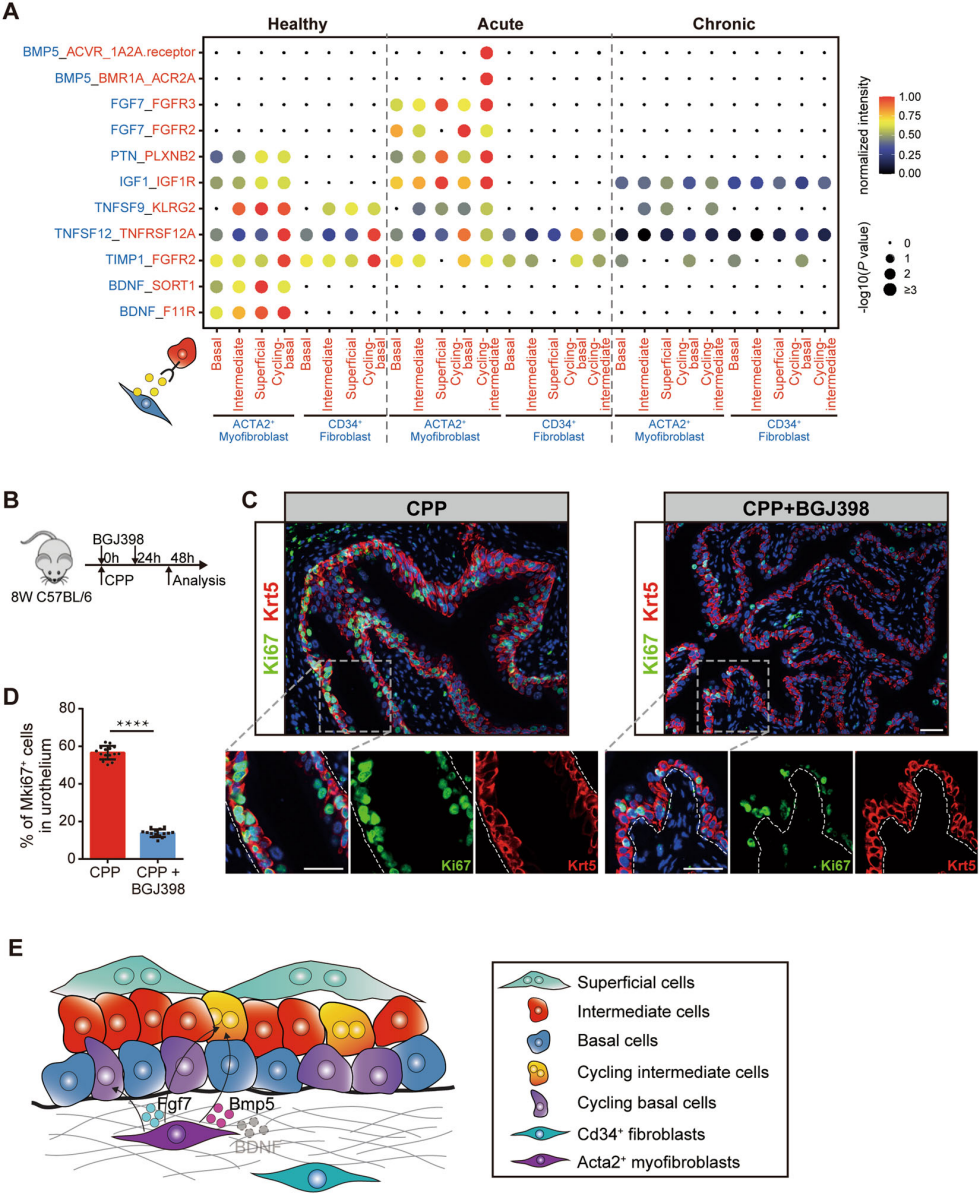
Cell-cell communication network regulates urothelial regeneration. **A** Dot plot shows specific ligand-receptor pairs between mesenchymal cells and epithelial cells in three conditions. **B** Schematic showing experimental design for assessing proliferating inhibition by Fgf-signaling blocking. **C** Immunostainings of Krt5 (in red) and Ki67 (in green) on sections of control (left) and BGJ398 treatment (right) CPP-injured bladder (scale bar 50 microns). **D** Bar plot shows the percentage of Ki67 positive cells in urothelial cells (*n* = 15 per condition, mean with SD, *t*-test, ****p < 0.0001). **E** Diagram of the main ligand-receptor interactions worked in bladder regeneration.

Having analyzed the heterogeneity of bladder mesenchymal cells, we next sought to determine whether bladder mesenchymal cells play a distinct role in urothelial regeneration. CellPhoneDB analysis was thus performed for two subsets of mesenchymal cells (myofibroblasts and fibroblasts) and different subsets of urothelial cells classified by basal/luminal phenotypes and cycling states. Interestingly, this analysis identified both condition-specific and cell type-specific communications. For example, Bdnf signaling was only enriched between myofibroblasts and urothelial cells in normal conditions (Fig. 6A). Bmp signaling has previously been reported to promote terminal differentiation of the urothelium^9^. Consistent with this, Bmp5 signaling were strongly activated between myofibroblasts and cycling intermediate cells in the acute injury condition, likely promoting the differentiation of cycling intermediate cells towards superficial cells (Fig. 6A). In the acute injury condition, we also detected strong Fgf7-Fgfr2/Fgfr3 signaling between myofibroblasts and urothelial cells (Fig. 6A). According to our previous NicheNet analysis (Fig. 4B), *Fgf7* might promote urothelial cell proliferation. We next tested the functional impact of Fgf7-Fgfr2/Fgfr3 during bladder regeneration in vivo by administering BGJ398, an inhibitor of Fgfr2 and Fgfr3 (Fig. 6B). This experiment showed that inhibition of Fgf7 signaling significantly decreased the proportion of cycling urothelium after injury (Fig. 6C, D), supporting a proliferation-promoting role of Fgf7-Fgfr2/Fgfr3 in bladder regeneration. Altogether, our data demonstrates that epithelial-myofibroblast crosstalk plays a critical role in urothelial homeostasis and repair and suggests that *Acta2*^*+*^*Car3*^*+*^ myofibroblasts function as a proliferation niche by producing Fgf7.

## Discussion

Although bladder urothelial injury patterns after CPP exposure have been widely examined in rodent models, this process is still poorly understood at the single-cell level. In this study, we report 50 404 single-cell transcriptomes of bladder mucosa under healthy, acute, and chronic conditions. We identified 23 transcriptionally distinct subpopulations of cells, including epithelial, mesenchymal, endothelial, macrophage, mural, and NK cells. Using this dataset, we unmasked the heterogeneity and lineage relationship of cycling urothelial cells. We also deciphered the niche mediating urothelial regeneration and identified a subset of *Acta2*^+^ myofibroblast cells that might play a critical role in urothelial regeneration.

Genetic lineage tracing studies and pulse-chase analyses suggest that both intermediate and basal cells can produce superficial daughters, depending on the conditions^5–11^. We previously identified a population of bi-nucleated intermediate cells and demonstrated that this population serves as the direct progenitor for superficial cell regeneration. Here, the findings of our single-cell trajectory analyses agree well with these observations (Fig. 3D and E). Our study thus provides fundamental insights into lineage relationships during urothelial cell regeneration. Moreover, the single-cell analysis also confirmed the cell cycle disparities between basal and intermediate cells. Our data suggests that the high expression of cell adhesion molecular genes in basal cells may be caused by the fact that basal cells undergo more complete divisions compared with intermediate cells (Fig. 3A–C and Fig. S3). Nevertheless, future studies in this area will be needed to determine the key factors that affect the division modes of urothelial progenitor subsets.

Tissue regeneration is guided by cell-cell communication between multiple cell types. In the bladder, stromal cells are critical for urothelial proliferation and differentiation^5,8,9,47,48^. However, a clear description of stromal cell types and cell-cell communication during bladder regeneration remains to be determined. Here, we found a group of myofibroblasts (*Acta2*^+^*Car3*^+^) that could have the most significant influence on urothelial cell proliferation (Fig. 6). Using immunofluorescence staining, myofibroblasts were found located near the epithelial layer (Fig. 5E). Single-cell analysis suggested that myofibroblasts significantly upregulated the expression of several signaling ligands, including *Bmp5*, *Igf*, and *Fgf7*, after acute injury. Notably, both Bmp and Fgfr signaling have been demonstrated to participate in urothelial cell regeneration^9,47,48^. In current study, we selected and validated Fgf7-Fgfr signaling using the Fgfr inhibitor BGJ398. Blocking Fgfr signaling significantly reduces the number of Ki67^+^ proliferating cells in the urothelial layer, verifying the predicted results of cell communication analysis (Fig. 6B–D). Further investigations of the roles of other crosstalk during bladder regeneration would be very intriguing. Moreover, the stromal cell analysis in this study showed that serial CPP treatment did not induce bladder fibrosis, as no fibroblast-myofibroblast transition, or ECM accumulation were observed under the chronic condition (Fig. 5F−H).

In summary, our study provides a valuable single-cell resource for deciphering the cellular heterogeneity of bladder mucosa after injury and has considerably improved our understanding of cellular mechanisms during bladder regeneration. In addition, our finding that myofibroblasts play an important role during chemically induced bladder regeneration may also shed light on exploring cellular mechanisms underlying chemotherapy resistance of bladder cancer.

## Materials and methods

### Animals

8-week wild-type (WT) C57BL mice were purchased from Charles River (Beijing, China) and maintained in the Specific-Pathogen-Free Animal Research Centre of Renji Hospital. The animal experiments were approved by the Animal Research Ethics Committee of Renji Hospital, School of Medicine, Shanghai Jiao Tong University. The group separation of animals was randomly and no blinding was done.

### Chemical Injury

Chemical injury of the bladder was induced by intraperitoneal injection of a CPP (Sigma-Aldrich, USA; catalog #C7397) solution in phosphate-buffered saline (PBS, 15 mg/ml). CPP was administered in two different protocols^19–21^. For the acute injury, 150 mg/kg CPP was administered. Bladders were collected 48 h after CPP injection. In the case of chronic injury, 75 mg/kg CPP was administered every third for a total of four injections. Bladders were harvested 48 h after the last round of injection. Each group of animals included males and females, and analysis was performed on at least *n* = 3 control and experimental animals for each condition.

### Fgfr-inhibitor treatment

BGJ398 (meilunbio, China; catalog # MB5516) was formulated as a suspension in PEG300/D5W (2:1, v/v) and administered orally 0 h before and 24 h after the CPP treatment at the dose of 20 mg/kg. Animals were sacrificed 24 h later.

### Immunofluorescence and antibodies

Bladders were embedded in paraffin and 5 μm sections were obtained. After deparaffinating, rehydrating and antigen retrieval, sections were blocked with 10% donkey serum for 1 h at room temperature. After blocking, specimens were incubated with the primary antibodies diluted in 1% donkey serum at 4 °C overnight. The next day, slides were washed with PBS 3 times for 10 min each and secondary antibodies were applied for 2 h at room temperature. The following primary antibodies and concentrations were used in these studies: mouse anti-E-Cadherin (1:300, BD #610181, USA), mouse anti-Ki67 (1:400, Cell signaling #9449, USA), rabbit anti-Ki67 (1:150, Abcam #ab15580, UK), mouse anti-CK20 (1:250, Dako #M7019, Denmark), rabbit anti-SPRR2F (1: 50, proteintech #24648-1-AP, USA), rabbit anti-UPK1A (1: 300, Sigma #HPA049879), mouse anti-p63 (1:100, Santa Cruz #sc-71826, USA), rabbit anti-CK5 (1:200, Abcam #ab52035), mouse anti-α-SMA (1:200, Arigo #SQab1735, China), rabbit anti-CD34 (1: 200, Abcam #ab81289). The following secondary antibodies and concentrations were used in our studies: Jackson ImmunoResearch (PA, USA) Alexa Fluor 488 donkey anti-rabbit IgG (711-545-152; 1:600), Alexa Fluor 488 donkey anti- mouse (715-545-150; 1:600), Alexa Fluor 594 donkey anti- rabbit IgG (711-585-152; 1:500), Alexa Fluor 594 donkey anti-mouse IgG (715-585-151; 1:500), Alexa Fluor 647-conjugated donkey anti-mouse IgG (715-605-150; 1:300).

### Imaging

All images were captured on Leica TCS SP5 inverted confocal (Leica, Germany) and Leica DM2500 (Leica). Image processing and cell counts were performed using ImageJ.

### Single-cell isolation and preparation of suspensions

Bladders were collected from normal adult mice, acute injured mice and chronic injured mice. The mucosal layer was obtained by the methods described previously with slight modifications^49^. The collected bladders were cut in half. The boundary between mucosa and detrusor was found under the microdissection microscope. Using one forceps to hold the detrusor, and the other to hold the mucosa, peel the mucosa away from the detrusor carefully. The mucosal layer was cut into pieces and enzymatically digested with collagenase IV (Gibco, USA; catalog # 17104019) and DNase I (Sigma; catalog # AMPD1) for 30 min at 37 °C, with agitation. After digestion, samples were sieved through a 70-μm cell strainer, washed with 1% BSA and 2 mM EDTA in PBS, and centrifuged for 8 min at 500×g. Single cell suspensions were run through Lympholyte-H separation (Cedarlane, Canada; catalog # CL5020) to remove RBCs and debris according to manufacturer’s specifications. Pelleted cells were then re-suspended in PBS with 1% BSA and assessed for viability and size using a Countess instrument (Thermo, USA).

### Single cell Library preparation and sequencing

To capture individual cells, we utilized the Chromium Single Cell 3’ Reagent Version 2 Kit (10X Genomics, USA). 6 000 cells were targeted for capture per sample. Then, cell suspension of each sample was run in the Chromium Controller with appropriate reagents to generate single cell gel bead-in-emulsions (GEMs) for sample and cell barcoding. Amplified cDNA and final libraries were evaluated on an Agilent BioAnalyzer using a High Sensitivity DNA Kit (Agilent Technologies, CA, USA). The libraries were then pooled and sequenced on NovaSeq 6000 (Illumina, CA, USA) at a depth of ~400 M reads per sample.

### scRNA-seq data preprocessing and quality control

Raw sequencing data were converted to FASTQ files with Illumina bcl2fastq, version 2.19.1 and aligned to the *Mus musculus* genome reference sequence (mm10). The Cell Ranger (10X Genomics, 2.1.1 version) analysis pipeline was used to sample demultiplexing, barcode processing and single-cell 3′gene counting to generate a digital gene-cell matrix from these data. Then, the gene expression matrix was processed and analyzed by Seurat package^50^. We performed Seurat-based filtering of cells based on the number of detected genes per cell (> 500) and the percentage of mitochondrial genes expressed (<10%). The mitochondrial genes and ribosomal genes were also removed from the gene expression matrix.

### Filtering cell doublets

Doublets of scRNA-seq were excluded by first using *DoubletFinder*^51^ and then estimating if our dataset included any clusters enriched for cell doublets based on the expression patterns of cell type–specific markers.

The basic information for single cell datasets of all samples is shown in Fig. S1A.

### Batch effect removing, dimensionality reduction, clustering and visualization

After quality control, SCTransform normalization was performed separately for each dataset. Since data from six samples were processed and sequenced in batches, batch number was used to remove potential batch effect. In this process, 3000 features which were selected by *SelectIntegrationFeatures* function were used to create potential Anchors with *FindIntegrationAnchors* function of Seurat. Subsequently, *IntegrateData* function was used to integrate data and create a new matrix with 3000 features, in which potential batch effect was regressed out.

To reduce the dimensionality of the scRNA-Seq dataset, principal component analysis (PCA) was performed on an integrated data matrix.

Clustering was then performed using graph-based clustering and visualized using t-SNE with Seurat function *RunTSNE* or UMAP with Seurat function *RunUMAP*.

### Cell type annotation

Conventional markers described in previous studies were used to categorize every cluster into a known biological cell type: urothelial cells (*Cdh1*, *Epcam* and *Gata3*), mesenchymal cells (*Vimentin*, *Sparc* and *Dcn*), endothelial cells (*Pecam1*), macrophage (*Cd68* and *Cd83*), mural cells (*Cspg4*) and NK cells (*Hcst* and *Ltb*).

### Estimation of basal/luminal identity of urothelium subsets

As urothelium usually been divided into basal, intermediate and superficial subsets by cytokeratin and uroplakins, we calculated the *Pearson* coefficient for gene expression of cytokeratin, uroplakins and other known markers such as *Itga6*, *Trp63* and *Foxa1* in urothelium. We determined two gene sets that can be used to identify luminal cells (intermediate and superficial cells) and basal cells respectively (Fig. S2A). Then we used the *AddModuleScore* function in the Seurat R package to score epithelial cells with the two gene sets^52,53^. Single cells were assigned to different cell types based on the maximum expression score.

### Estimation of proliferation status

To measure the proliferation status of single cell, we scored cells using a set of characteristic genes involved in cell-cycle including 43 G1/S and 54 G2/M cell cycle genes^54^. Cycling cells were defined to be the ones with high G1/S score or G2/M score, and non-cycling cells were the ones with low G1/S and G2/M scores. Data-derived thresholds of 2 MADs above the median were used to decide whether a score is high or low as previously described^55^.

### Differential gene expression analysis

Differentially expressed genes (DEGs) were determined with the *FindMarkers* / *FindAllMarkers* function from the Seurat package (one-tailed Wilcoxon rank sum test, *p*-values adjusted for multiple testing using the Bonferroni correction). For computing DEGs, all genes were probed provided they were expressed in at least 10% of cells in either of the two populations compared and the expression difference on a natural log scale was at least 0.1.

### Pseudotime trajectory analysis by monocle3

Monole3^31–33^ was performed on urothelial cells from three conditions to uncover the pseudotime trajectory of bladder epithelium. Dimensionality reduction and trajectories was done by standard workflow and default parameters. For both branch1 and branch2, we set node in S phase as the root node to calculate the pseudo time using the *order_cells* function.

To draw the kinetics plot of G2M-score of cycling basal cells and cycling intermediate cells across pseudo time in Fig. 3H, we choose cells which pseudo time less than 3.5, as all basal cells and more than 95% intermediate cells are in this range, and the pseudo time of over 90% superficial cells are bigger than 3.5.

### Inferring cell state transition by RNA velocity

RNA velocities were predicted using scVelo in python program^56^. Briefly, spliced/unspliced reads were annotated by velocyto.py with CellRanger (version 2.2.0), generating BAM files and an accompanying GTF; they were then saved in.loom files. The.loom files were loaded to the scvelo python pipeline using *scv.read* function and they generated count matrices for spliced and unspliced reads. Next, the count matrices were size-normalized to the median of total molecules across cells. The top 3 000 highly variable genes were selected out of those that pass a minimum threshold of 10 expressed counts commonly for spliced and unspliced mRNA. Considering that the assumptions of a common splicing rate and the observation of the full splicing dynamics with steady-state mRNA levels were often violated, we used the function *recover_dynamics*, a likelihood-based dynamical model, to break these restrictions. Finally, the directional flow was visualized as streamlines in the UMAP embedding.

To quantify the cell state transition probability, we calculated the velocity-based cell transition matrix by the *transition_matrix()* function from scvelo, of which the element was the *Pearson* correlation coefficient between the velocity vector and cell state difference vectors of the column cell. We defined the destination of a cell by identifying the highest correlation value.

### SCENIC analysis

Active TFs and their gene targets in the mesenchymal cells were inferred using python package “pySCENIC”^57^. The co-expression modules were run by GRNBoost (SCENIC version 0.1.5). The motifs database for *Mus musculus* was downloaded from the website https://pyscenic.readthedocs.io/en/latest/. The input gene matrix was the non-normalized gene matrix of mesenchymal cells. The activity of the regulons is scored and binarized with AUCell, which effectively determines whether the genes in each regulon are enriched in each cell using the distribution of regulon activity across all cells in the dataset.

### Pathway analysis

GO enrichment analysis was performed on DEGs using clusterProfiler^58^ with pvalueCutoff = 0.01 and qvalueCutoff = 0.05 as the cut-off criteria.

### Cell–cell communication analysis with CellPhoneDB 2

We applied CellPhoneDB^15,34^ to our data to infer ligand-receptor interactions present between urothelium and non-urothelial cells (mesenchymal cells, endothelial cells, macrophages, NK cells and mural cells, for Fig. 4), as well as epithelial subsets and mesenchymal subsets (basal, intermediate, superfacial, cycling basal, cycling intermediate, *Cd34*^+^ fibroblast and *Acta2*^+^ myofibroblast, for Fig. 6). Cells from six samples were clustered by cell type and conditions. Interaction pairs have P-values < 0.05 returned by CellPhoneDB, were selected for the evaluation of relationships between cell types. The mice gene were transformed to human gene using Biomart, and non-log-transformed UMI counts were used as the expression values for receptors and ligands.

### NicheNet analysis

We applied NicheNet^35^ to predict the transcriptional influence on cell cycle entry of ligands within stromal cells. Epithelial cells were defined as receiver population, and mesenchymal cells, endothelial cells, macrophages, mural cells and NK cells were defined as sender populations. Gene sets of interest were defined based on differential gene expression analysis of cycling urothelium (avg_logFC > 0.25; expressed in at least 10% of cycling urothelium) and purified by intersecting with cell cycle-related gene list (downloaded from Ensembl BioMart). The detailed interest gene set was shown in Table S3. A background gene set includes all other genes that were not differentially expressed between these populations. NicheNet’s ligand-target model was converted from human to mouse genes using the *convert_human_to_mouse_symbols* function. Activities of ligands identified in our CellPhoneDB analysis were ranked by calculating *Pearson*’s correlation coefficients of the ligand-target regulatory potential scores for each ligand and the target indicator vector, which defines a gene as present or absent within the gene set of interest.

### Quantification and statistical analysis

Statistical analysis was performed using R (version 3.4.3) and GraphPad Prism (GraphPad Software Inc., CA, USA; version 7.04). Detailed descriptions of statistical tests are specified in the results section and in the Figure legends.

## Supplementary information

Supplementary Figure legends

Fig.S1 Single-Cell RNA-Seq data features and cell type annotation.

Fig.S2 Heterogeneity of urothelial cells.

Fig.S3 Cell cycle difference between basal and intermediate cells

Table S1

Table S2

Table S3

Table S4

## Data Availability

All relevant data are available from the authors. Single-cell RNA-seq are available in the Short Read Archive under accession number GSE164557.

## Code availability

This study did not generate any unique code. All code or software tools used in this study follows the official tutorial and are freely or commercially available.
