# Supplementary material for: Single-cell analysis reveals urothelial cell heterogeneity and regenerative cues following cyclophosphamide-induced bladder injury
Source: Cell Death Dis. 2021 May 5;12(5):446. doi: 10.1038/s41419-021-03740-6 (PMC8099875; doi:10.1038/s41419-021-03740-6)
Supplement: Supplementary file 1 — Supplementary Figure legends [file 41419_2021_3740_MOESM1_ESM.docx]

**Supplementary Figure 1. Single-Cell RNA-Seq data features and cell type annotation.** (A) Table of sample information. (B) Violin plots show number of UMIs (left) and genes (right) detected in each sample were similar. (C) Feature plots show expression of signature genes for each cell type. Cells are colored by gene expression.

**Supplementary Figure 2. Heterogeneity of urothelial cells.** (A) Heatmap shows the *Pearson* coefficient for gene expression of cytokeratin, uroplakins and other known markers such as *Itga6*, *Trp63* and *Foxa1* in urothelium. (B) UMAP visualization colored by basal and luminal scores. (C) Bar plot show the cell proportion of cells under each condition in each cluster.

**Supplementary Figure 3. Cell cycle difference between basal and intermediate cells.** (A) Scatter plot show G2/M score of cycling basal and cycling intermediate subset. Cells are colored by G2/M score and shaped by cell type. (B) Expression of genes linked to cell adhesion in each epithelial subset.
