# Supplementary figures and images for: Single-cell analysis reveals urothelial cell heterogeneity and regenerative cues following cyclophosphamide-induced bladder injury
Source: Cell Death Dis. 2021 May 5;12(5):446. doi: 10.1038/s41419-021-03740-6 (PMC8099875; doi:10.1038/s41419-021-03740-6)

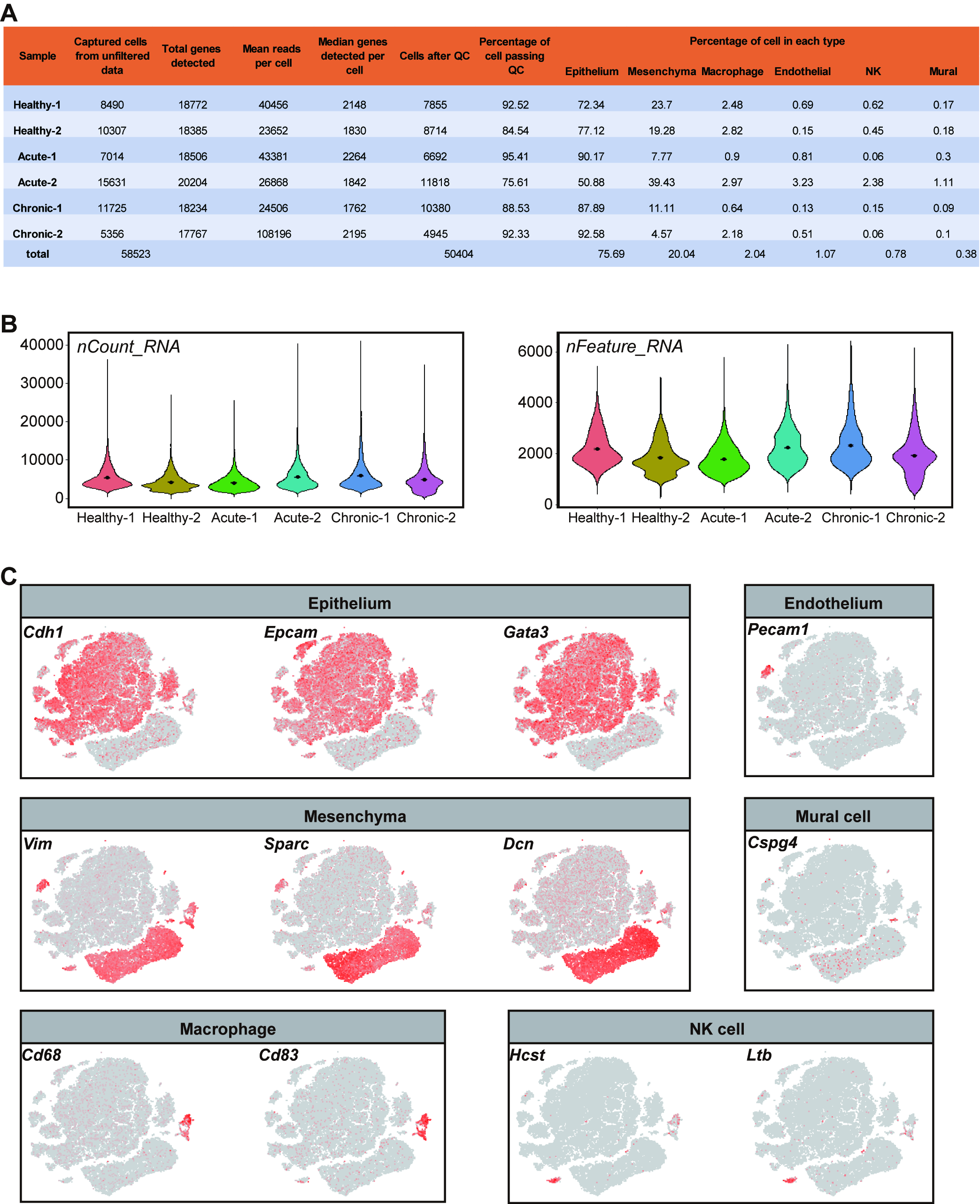

Supplement: Supplementary file 2 — Fig.S1 Single-Cell RNA-Seq data features and cell type annotation. [file 41419_2021_3740_MOESM2_ESM.tif]

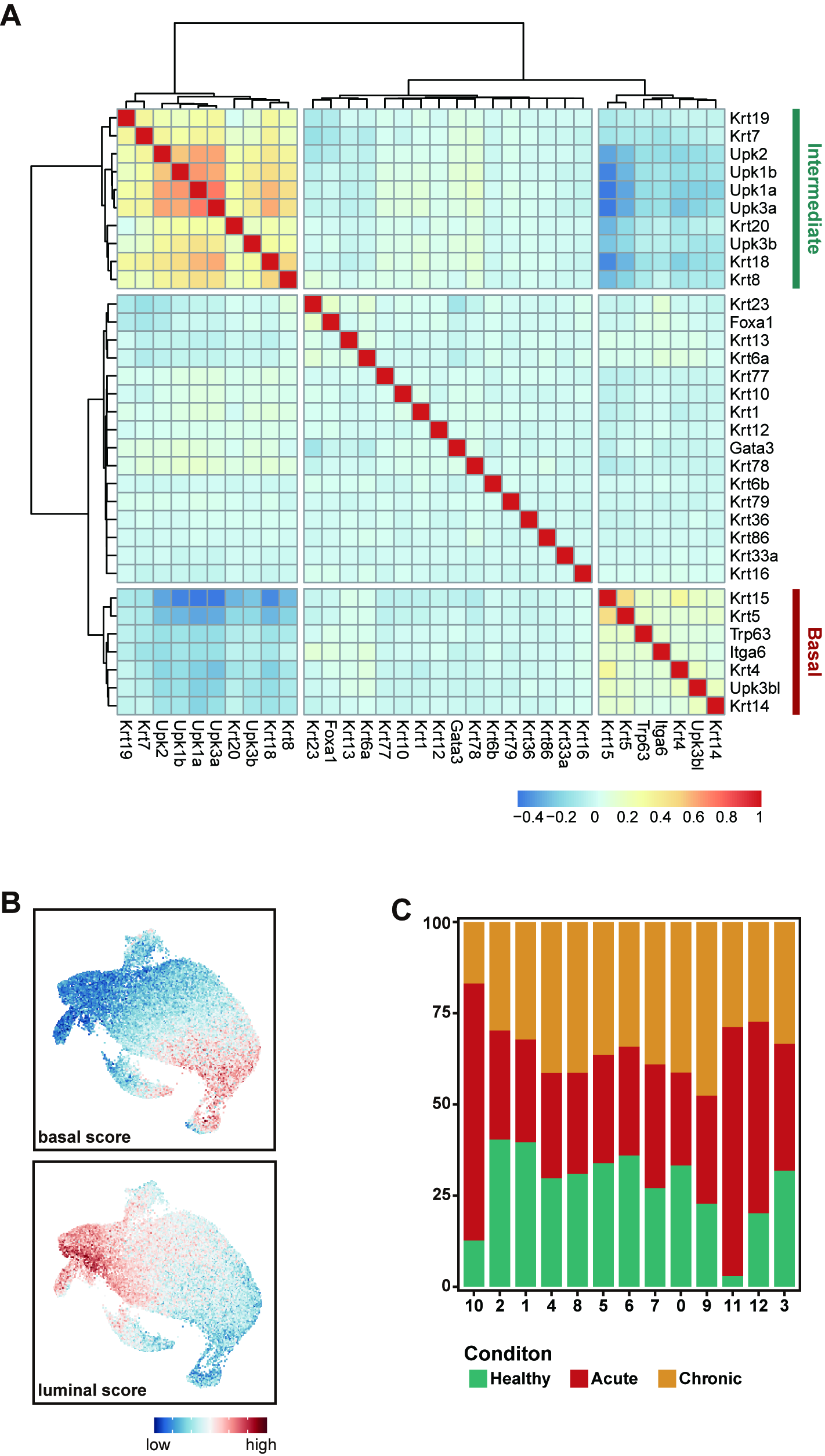

Supplement: Supplementary file 3 — Fig.S2 Heterogeneity of urothelial cells. [file 41419_2021_3740_MOESM3_ESM.tif]

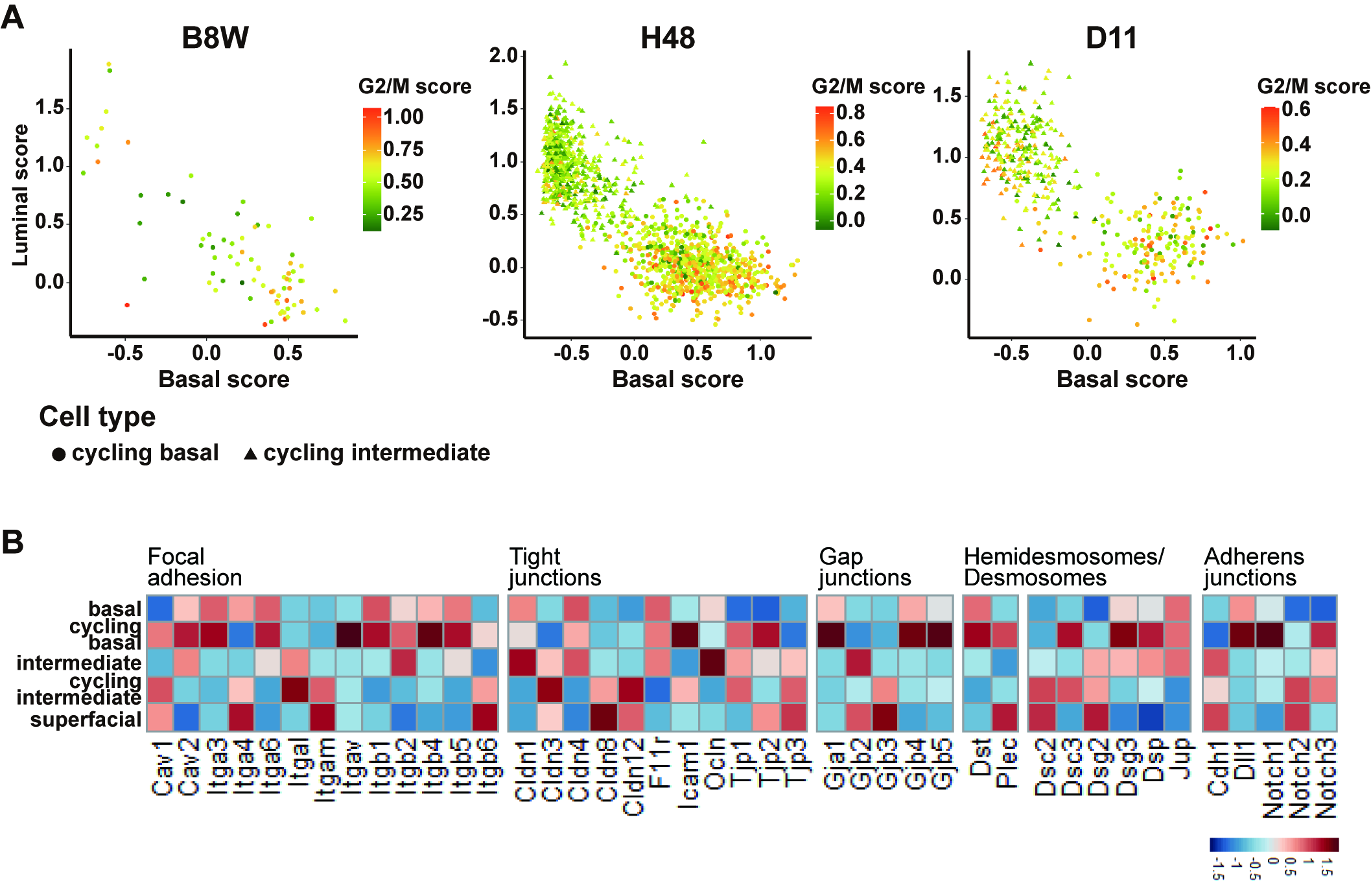

Supplement: Supplementary file 4 — Fig.S3 Cell cycle difference between basal and intermediate cells [file 41419_2021_3740_MOESM4_ESM.tif]
